# Referring patients with chronic kidney disease back to primary care: a criteria-based analysis in outpatient renal clinics

**DOI:** 10.1186/s12882-021-02367-1

**Published:** 2021-05-19

**Authors:** Carola van Dipten, Davy Gerda Hermina Antoin van Dam, Wilhelmus Joannes Carolus de Grauw, Marcus Antonius Gerard Jan ten Dam, Marcus Matheus Hendrik Hermans, Willem Jan Jozef Assendelft, Nynke Dorothea Scherpbier-de Haan, Jacobus Franciscus Maria Wetzels

**Affiliations:** 1 Department of Primary and Community Care, Radboud Institute for Health Sciences, Radboud university medical center, Geert Grooteplein Noord 2, postal route 117, 6525 EZ Nijmegen, The Netherlands; 2 Department of Internal medicine, Viecuri Medical Center, Tegelseweg 210, 5912 BL Venlo, The Netherlands; 3 Department of Internal medicine, Canisius Wilhelmina Hospital, Weg door Jonkerbos 100, 6532 SZ Nijmegen, The Netherlands; 4 Department of Nephrology, Radboud Institute for Health Sciences, Radboud university medical center, Geert Grooteplein Zuid 8, postal route 464, 525 GA Nijmegen, The Netherlands

**Keywords:** Chronic kidney disease, Shared care, Back referral, Primary care, Retrospective cohort study

## Abstract

**Background:**

The increased demand for nephrology care for patients with chronic kidney disease (CKD) necessitates a critical review of the need for secondary care facilities and the possibilities for referral back to primary care. This study aimed to evaluate the characteristics and numbers of patients who could potentially be referred back to primary care, using predefined criteria developed by nephrologists and general practitioners.

**Method:**

We organised a consensus meeting with eight nephrologists and two general practitioners to define the back referral (BR) criteria, and performed a retrospective cohort study reviewing records from patients under nephrologist care in three hospitals.

**Results:**

We reached a consensus about the BR criteria. Overall, 78 of the 300 patients (26%) in the outpatient clinics met the BR criteria. The characteristics of the patients who met the BR criteria were: 56.4% male, a median age of 70, an average of 3.0 outpatients visits per year, and a mean estimated glomerular filtration rate of 46 ml/min/1,73m^2^. Hypertension was present in 67.9% of this group, while 27.3% had diabetes and 16.9% had cancer. The patients who could be referred back represented all CKD stages except stage G5. The most common stage (16%) was G3bA2 (eGFR 30 ≤ 44 and ACR 3 ≤ 30).

**Conclusion:**

A substantial proportion of patients were eligible for referral back to primary care. These patients often have a comorbidity, such as hypertension or diabetes. Future research should focus on generalisability of the BR criteria, the feasibility of actual implementation of the back referral, follow-up assessments of renal function and patient satisfaction.

## Background

Most patients with chronic kidney disease (CKD) have a mild to moderate CKD (stages 1–3), as classified according to the Kidney Outcomes Quality Initiative guidelines [1, 2]. Consequently, most patients with CKD can be treated by their general practitioner (GP). The Dutch CKD guideline for primary care [3], comparable to the National Institute for Health and Care Excellence guidelines in the UK [4], provides GPs with tools for diagnostics, monitoring and treatment. Also, this guideline clearly defines the criteria for consultation with, or referral to, secondary care providers; for instance, in the case of a rapid decline in the glomerular filtration rate or severe proteinuria, the GP should refer the patient to, or at least consult, a nephrologist. Timely referrals have been repeatedly shown to affect outcome measures, such as mortality and reaching end-stage renal failure [5], and are essential when a requirement for renal replacement therapy is indicated.

The rising prevalence and incidence of CKD will increase the number of referrals made to nephrology practitioners [6–8], increasing healthcare costs and posing a burden for both patients and nephrologists [9, 10]. However, it is questionable whether many of the referred patients with CKD actually need long-term care from a nephrologist. Patients with stable CKD could be referred back to primary care with the proper support [4]. The increased demand for hospital care necessitates a periodical, broadly supported, criteria-based critical review of patients in secondary care facilities, together with an evaluation of the possibilities for referral back to primary care where possible.

Remarkably, none of the above-mentioned guidelines clearly indicate when patients can or should be referred back to primary care, nor, to the best of our knowledge, have any studies investigated the proportion and characteristics of patients who might be eligible. The aim of this study was therefore to develop criteria for referral from secondary care back to primary care, and to evaluate the characteristics and the number of patients who could be eligible for back referral.

## Materials and methods

### Development of criteria for referral back to primary care

To define the back referral criteria (BR criteria) for discharging patients with CKD to primary care, we organised a consensus meeting with eight nephrologists from different hospitals in the south-east of the Netherlands and two GPs, both members of the national CKD guideline committee (see names and hospitals or practices in acknowledgements and author contributions). In this meeting, we also defined which patients could not be referred back to primary care. A summary of the meeting was circulated among the participants for feedback, and a consensus document was made. The BR criteria were returned to all participants for checking, and were accepted by all.

### Proportion and characteristics of patients who fulfilled the BR criteria

#### Study setting

In a retrospective cohort study, we reviewed the records of patients under nephrologist care in three hospitals (VieCurie Medical Center (VMC) in Venlo, and Canisius Wilhelmina Hospital (CWH) and Radboud university medical center (RUMC) in Nijmegen). We recruited participants at the outpatient clinics because hospitalized patients are by definition not stable. Patients were included for their kidney disease, regardless of the aetiology. Also, patients requiring follow-up after hospitalization, patients with a history of acute kidney injury or previous kidney transplantation were included. Patients visiting the renal clinics were informed about the study, and their informed consent was obtained prior to their participation. There were no exclusion criteria. Patients were recruited by all nephrologists in the participating renal clinics. The recruitment period lasted until about 100 patients per clinic were included. This study is an exploration of back referral criteria and the number of potential patients eligible for referral back. For this, the patients were not actually referred back to primary care.

#### Data collection and analysis

The researchers (CvD, resident in general practice and PhD candidate in CKD in primary care, and DvD, resident in internal medicine) extracted the patient demographics and clinical data from the medical records. The authorised web-based system Castor was used for data collection and storage. This system enables researchers to build electronic Case Report Forms and store data [11]. The researchers completed a standard form for each patient, then checked whether the patient met the BR criteria. A patient’s nephrologist was consulted by the researchers in the following cases: doubts about the back referral; a lack of clarity in the patient’s medical file; if the CKD was stable but was caused by a specific nephrological disease; and when the researchers found notes about a back referral in the medical file despite the patient not meeting the BR criteria. We used descriptive statistics (SPSS version 25) to assess the patients’ characteristics and the proportion of patients with CKD who could be referred back to primary care. We used chi-square tests and independent-sample *t*-tests to assess the statistical significance of the results. In the case of non-normal distributions, we used Mann Whitney U-tests to evaluate statistical significance. A *P*-value < 0.05 was considered significant.

## Results

The following BR criteria were defined:

- Patients with stable kidney function **AND** stable blood pressure **AND** stable metabolic parameters, in whom renal replacement therapy is not expected within five years (for definitions, see textbox).
- Patients with haematuria and/or proteinuria less than 1 g/24 h **AND** with stable kidney function **AND** stable blood pressure. Exception: patients with IgA nephropathy who are eligible (now or in the future) for immunosuppressive therapy.
- Patients not eligible for renal replacement therapy (because of comorbidity, age or patient preference), provided that the patient’s kidney function and metabolic parameters are stable.

**Table:** 

*Stable kidney function*: a decrease in estimated glomerular filtration rate (eGFR) less than	
1a) 25% compared to the first measurement within five years **OR** 1b) 5 ml/min/1.73 m^2^/year	
**AND**	
2) proteinuria less than 0.5 g in a 24-h urine collection in case of glomerular haematuria and less than 1 g per 24 h in the absence of haematuria.	
*Stable blood pressure:* patients with a stable blood pressure at or below their individual target value and without drug adjustments in the last two consultations.	
*Stable metabolic parameters:* patients without medication changes concerning phosphate, calcium, parathormone or haemoglobin in the last two consultations.	

In addition, we defined the criteria for patients who should not be referred back to primary care:

- Patients with a rapid decrease of kidney function, defined as a drop in eGFR of more than 5 ml/min/1.73m^2^/year **OR** a decrease of more than 25% in five years.
- Patients with proteinuria of more than 1 g/24 h.
- Patients with an eGFR less than 30 ml/min/1.73m^2^, showing progression and with reasonable to good physical health, who would be eligible for renal replacement therapy.
- Patients expected to require renal replacement therapy within five years.
- Patients using immune suppressive drugs in the context of a renal disease or after a kidney transplant.
- Patients with specific nephrological diseases with a risk of relapse or a high risk of disease progression, such as patients with polycystic kidney disease or patients with lupus nephritis. Referral back to primary care is only considered possible in patients with stable kidney disease, five years after stopping immunosuppressive therapy.
- Patients with multimorbidity and/or metabolic complications which cannot be regulated in primary care and/or for which medication shifts have occurred within the last two consultations.
- Patients with CKD not amenable to palliative therapy in primary care due to difficulties in symptom regulation.

### Study population and patients fulfilling the BR criteria

The study was conducted between February and September 2018. We included a total of 300 patients: 102 patients from CWH, 100 patients from VMC and 98 patients from RUMC. Of those, 57.5% were male, the median age was 67.5 years and the average number of outpatient visits was 3.8 times a year. The average eGFR was 42 ml/min/1.73m^2^, and most patients had moderate (40.9%) to severe (43.2%) albuminuria. Hypertension (60%), diabetes mellitus (24.7%) and cancer (unspecified) (19.7%) were the most common comorbidities. Glomerular pathology was the most common aetiology for CKD (see Table 1).

**Fig. 1:**
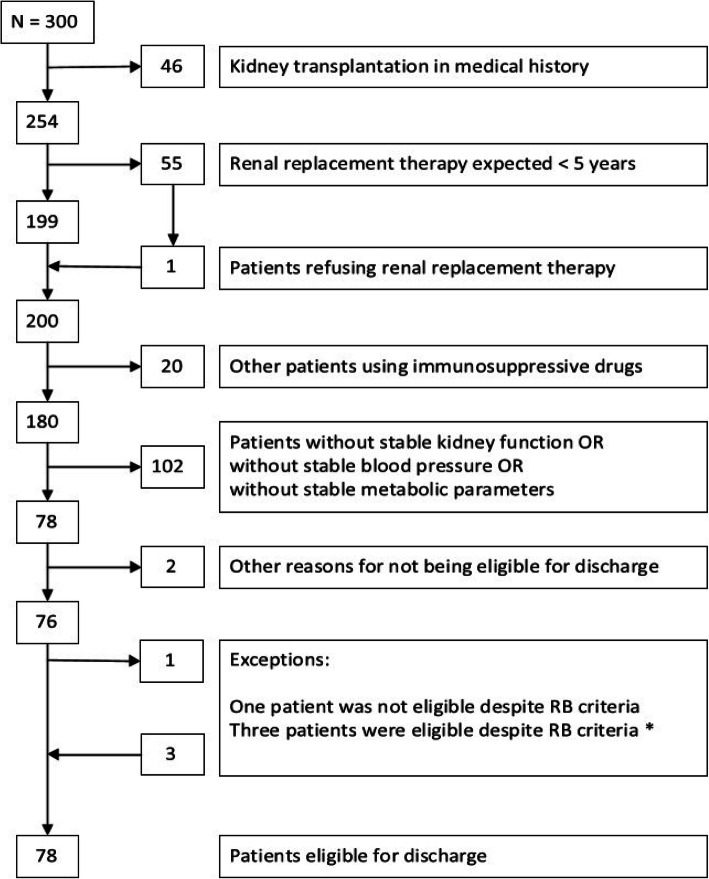
Algorithm of patients selected for referral back (BR) to primary care. The patients not meeting the BR criteria are removed on the right-hand side. *Exceptions: - In one case, the nephrologists were worried about the risk of renal decline after an episode of acute kidney injury, despite the patient’s CKD stability, and advised the continued follow up of this patient at the renal clinic. - Three patients did not strictly meet the BR criteria, but were still eligible for referral back; two patients had more severe proteinuria, which was stable for over five years, while one patient had recently started blood pressure medication, but the low complexity of their case allowed their referral back to primary care

Overall, 78 of the 300 patients (26%) in the outpatient clinics met the BR criteria (see Fig. 1), varying between 23 and 30% per clinic. Of the patients who met the BR criteria, 56.4% were male, and their median age was 70 years (see Table 1). Their average monitoring frequency was lower (3.0 vs. 4.1 times per year, *P* = 0.000) and their mean eGFR was higher (46 vs. 40 ml/min/1.73m^2^, *P* = 0.044) than the patients who did not meet the BR criteria. Patients who met the BR criteria more often had heart failure (19.2% vs. 7.8%, *P* = 0.007) and had suffered a transient ischaemic accident (TIA) or a cerebrovascular accident (CVA) (21.8% vs. 10.8%, *P* = 0.018), but less commonly had cancer (16.9% vs. 20.8%, *P* = 0.042). Patients eligible for referral back to primary care were classified into all CKD stages, most commonly into stages G3bA2 (16%) and G4A2 (14.1%) (see Table 2).

## Discussion

### Summary of the main findings

When applying the BR criteria to the records of patients at the renal clinics, the proportion of patients with CKD who could be referred back to primary care varied between 23 and 30% per clinic. In addition, several patients seemed to be potential candidates for a future back referral, such as patients with stable CKD but who currently had too short of a follow-up period, or patients who had recently changed their blood pressure medication because of side effects rather than problems reaching their target values. The criteria were easily applicable and their use required little discussion. In only four cases, the judgments of the researchers and nephrologists overruled the BR criteria (for more detail see Fig. 1). Patients eligible for referral back to primary care represented all CKD stages except for stage G5, and did not differ much from the patients not eligible for back referral in terms of their demographics.

**Table 1:** Characteristics of all patients in the renal clinics, and a comparison between those who were and were not considered eligible for referral back (BR) to primary care.

	All patients *N* = 300	Patients meeting the BR criteria *N* = 78 (26%)	Patients not meeting BR criteria *N* = 222 (74%)	P-value
Number of patients per hospital	CWH: 102/300 (34.0%)	CWH: 31/102 (30.4%)	CWH: 71/102 (69.6%)	
	VMC: 100/300 (33.3%)	VMC: 23/100 (23.0%)	VMC: 77/100 (77.0%)	
	RUMC: 98/300 (32.7%)	RUMC: 24/98 (24.5%)	RUMC:74/98 (75.5%)	
Demographics patients				
Gender, male (%)	173 (57.5%)	44 (56.4%)	129 (58.1%)	0.794
Age in years, median (range)	67.5 (19–96)	70 (25–88)	67 (19–96)	0.265
Number of outpatient visits, mean	3.8 ± 1.66 (*n* = 259)	3.0 ± 1.28 (*n* = 76)	4.1 ± 1.69 (*n* = 183)	< 0.001
Aetiology				
ADPKD*	33/300 (11.0%)	7/78 (9.0%)	26/222 (11.7%)	0.506
Glomerular diseases^1^	82/300 (27.3%)	18/78 (23.1%)	64/222 (28.8%)	0.327
Systemic diseases^2^	20/300 (6.7%)	0/78 (0.0%)	20/222 (9.0%)	0.006
Tubulointerstitial nephritis	11/300 (3.7%)	2/78 (2.6%)	9/222 (4.1%)	0.547
Drug-induced CKD	7/300 (2.3%)	2/78 (2.6%)	5/222 (2.3%)	0.875
Vascular CKD	61/300 (20.3%)	19/78 (24.4%)	42/222 (18.9%)	0.304
Diabetic nephropathy	26/300 (8.7%)	8/78 (10.3%)	18/222 (8.1%)	0.562
Other cause^3^	38/300 (12.7%)	11/78 (14.1%)	27/222 (12.2%)	0.658
Unknown cause	20/300 (6.7%)	11/78 (14.1%)	9/222 (4.1%)	0.002
Measurements				
eGFR (ml/min/1.73^2^), mean	42 ± 21.07	46 ± 19.95	40 ± 21.3	0.044
Stage proteinuria, (%)				0.007
A1	47/296 (15.9%)	15/78 (19.2%)	32/218 (14.7%)	
A2	121/296 (40.9%)	41/78 (52.6%)	80/218 (36.7%)	
A3	128/296 (43.2%)	22/78 (28.2%)	106/218 (48.6%)	
Haemoglobin (g/dl), mean	13.21 ± 1.79 (*n* = 253)	13.54 ± 1.82 (*n* = 72)	13.05 ± 1.77 (*n* = 181)	0.054
Potassium (mmol/L), mean	4.5 ± 0.49 (*n* = 265)	4.4 ± 0.39 (*N* = 78)	4.5 ± 0,53 (*n* = 187)	0.865
Phosphate (mmol/L), mean	1.05 ± 0.28 (*n* = 224)	1.01 ± 0.29 (*n* = 63)	1.06 ± 0.27 (*n* = 161)	0.196
Systolic blood pressure (mm Hg), mean	131 ± 16.6 (*n* = 283)	131 ± 16.49 (*n* = 76)	131 ± 16.7 (*n* = 207)	0.967
Diastolic blood pressure (mm Hg), mean	73 ± 9.96 (*n* = 283)	74 ± 8.37 (*n* = 76)	73 ± 10.48 (*n* = 207)	0.424
Comorbidity				
Angina pectoris	33/271 (12.2%)	10/77 (13.0%)	23/194 (11.9%)	0.797
Myocardial infarction	42/272 (15.4%)	16/78 (19.8%)	26/194 (13.4%)	0.142
Heart failure	30 /270 (11.1%)	15/78 (19.2%)	15/192 (7.8%)	0.007
Hypertension (K86.87)	180/274 (65.7%)	53/78 (67.9%)	127/196 (64.8%)	0.620
TIA or CVA	38/272 (14.0%)	17/78 (21.8%)	21/194 (10.8%)	0.018
Hemiplegia	0/271 (0%)	0/78 (0%)	0/193 (0%)	NA
Peripheral vascular disease	41/270 (15.2%)	9/77 (11.5%)	32/193 (16.6%)	0.312
Diabetes mellitus	74/270 (27.4%)	21/77 (27.3%)	53/193 (27.5%)	0.608
*Uncomplicated*	35/270 (13.0%)	8/77 (10.4%)	27/193 (14.0%)	
*Complicated*	39/270 (14.4%)	13/77 (16.9%)	26/193 (13.5%)	
COPD	20/272 (7.4%)	6/78 (7.7%)	14/194 (7.2%)	0.892
Dementia	0/272 (0%)	0/78 (0%)	0/194 (0%)	NA
Liver disease	6/270(2.3%)	0/78 (0%)	6/192 (3.1%)	0.288
*Mild*	5/270 (1.9%)	0/78 (0%)	5/192 (2.6%)	
*Moderate / severe*	1/270 (0.4%)	0/78 (0%)	1/192 (0.5%)	
HIV or AIDS	1/271 (0.3%)	0/77 (0%)	1/194 (0.5%)	0.528
Cancer	53/269 (19.7%)	13/77 (16.9%)	40/192 (20.8%)	0.042
*Solid tumour*	51/269 (19%)	11/77 (14.3%)	40/192 (20.8%)	
*Metastatic*	2/269 (0.7%)	2/77 (2.6%)	0/192 (0%)	
Haematological malignancy	4/269 (1.5%)	0/78 (0%)	4/191 (2.1%)	0.198
Connective tissue disease	21/273 (7.7%)	4/77 (5.2%)	17/196 (8.7%)	0.332
Peptic ulcer	6/271 (2.2%)	2/77 (2.6%)	4/194 (2.1%)	0.787
Charlson Comorbidity Index, median (range)	5.3 (0–15) (*n* = 219)	5.3 (0–15) (*n* = 69)	5.3 (0–12) (*n* = 150)	0.938
Medication				
Angiotensin receptor blockers	186/272 (68.4%)	50/78 (64.1%)	136/194 (70.1%)	0.336
Diuretics	92/269 (34.2%)	25/76 (32.9%)	67/193 (34.7%)	0.777
Beta blockers	130/272 (47.8%)	38/78 (48.7%)	92/194 (47.4%)	0.847
Calcium channel blockers	105/272 (38.6%)	25/78 (32.1%)	80/194 (41.2%)	0.159
Vitamin D / alfacalcidol	151/271 (55.7%)	39/78 (50.0%)	112/193 (58.0%)	0.228
Erythropoeitin	19/272 (6.3%)	4/78 (5.1%)	15/194 (7.7%)	0.446
Phosphate binders	10/272 (3.7%)	0/78 (0%)	10/194 (5.2%)	0.041
Immunosuppressive drugs	67/300 (22.3%)	0/78 (0%)	67/222 (30.2%)	< 0.001

^1^ Glomerular diseases include: glomerulonephritis, nephrotic syndrome (any cause), glomerular proteinuria or haematuria (without biopsy), IgA nephropathy

^2^ Systemic diseases include: systemic lupus erythematosus, vasculitis, sarcoidosis

^3^ Other causes include: postrenal cause, renal artery stenosis, cancer (treatment)

± = standard deviation

.

**Table 2:**
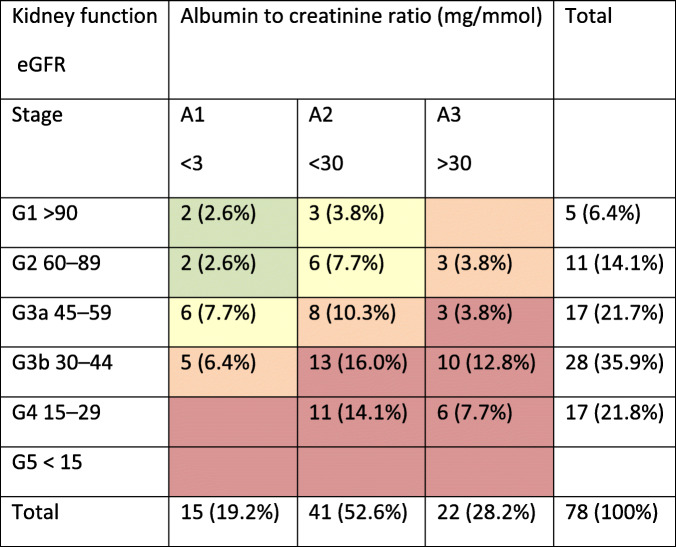
Patients eligible for referral back to primary care, classified by stage.

Different colours denote different levels of risk for cardiovascular events, progression to end-stage renal failure and mortality: green: low risk; yellow: moderately increased risk; orange: high risk; red, very high risk Classification according to the Kidney Outcomes Quality Initiative guidelines (KDOQI).

### Prerequisites for implementation in practice

A number of conditions must be met before patients can be referred back to primary care. First of all, the quality of care (QoC) for patients with CKD in primary care must be guaranteed. Some studies indicated that the QoC for these patients received solely from a GP is suboptimal [12, 13], while others have shown that GPs deliver an appropriate quality of care for patients referred back to primary care, in terms of periodic renal monitoring, preventing the deterioration of kidney function and maintaining blood pressure within an appropriate range [14, 15]. GPs recognition of patients with CKD in primary care and receiving co-management assistance from nephrologists, are both factors associated with improved QoC [13]. Our previous study shows that not all patients indicated for nephrologist care, actually receive nephrologist consultation or co-management. In addition, for patients aged 80 and older, it was questionable whether management by nephrologists contributes to a better clinical outcome [16]. To enhance the QoC in primary care, we believe it would be helpful to embed CKD care within the care of other chronic conditions to guarantee a periodic follow up [17]. Furthermore, to overcome other doubts about QoC in the primary care setting, we recommend the provision of care according to a shared care model. The co-management of patient care by nephrologists and GPs led to the increased monitoring of eGFR, urine albumin and metabolic parameters, and resulted in more prescriptions of angiotensin-converting enzyme inhibitors and angiotensin receptor blockers [18]. Shared care models for chronic conditions are predominantly found to have a positive effect [19–21]. Combining shared care with information technology such as a pop-up in medical records for recognising CKD, decision support in medical records or a web-based consultation system could also enhance QoC [22, 23].

Second, for the interdisciplinary management of patients, it is essential to make agreements on the tasks, responsibilities and communication between GPs and nephrologists. Not all professionals in the field will have the same expectations, as even among nephrologists there are different views about various tasks [24]; therefore, agreements should preferably be made regionally to ensure the close communication of GPs and nephrologists and to make use of a pre-existing collaborative relationships. A shared clinical information system could also facilitate better communication between primary and secondary care [25]. The current literature reveals that both GPs and nephrologists prefer shared care [26, 27], but only when sufficient information exchange is provided [24, 28]. GPs have said that, besides conservative care for older and frail patients with advanced CKD, they have little experience in treating patients with advanced CKD and welcome guidance from nephrologists [29]. Several studies have already been executed to improve co-ordination between primary and secondary care; for example, researchers introduced a kidney failure risk equation, which is a predictive model to determine whether care by a nephrologist is required, based on the patient’s risk for kidney failure [30, 31]. A predictive model alone is not sufficient for the co-ordination of CKD care between primary and secondary practices, however our BR criteria could also contribute to personalised decision making.

Last but not least, it is important to know whether patients are willing to be referred back to primary care. Not much has been written about this, despite patient willingness being a prerequisite for back referral. One study found that patients prefer to receive care from their GP, provided that GPs have access to a specialist for consultation or a system in which diagnostic procedures are organised by a specialist with patients subsequently being referred back to the GP for care [26]. Patients views and experiences concerning CKD care still need to be explored in more detail.

### Advice for further research

Future research should focus on the actual implementation of the referral of patients with CKD back to primary care. The enabling and disabling factors need to be further explored, taking into account the opinions of patients, GPs and nephrologists. We didn’t study the underlying motives for push and pull factors among GPs and specialists. This also would require additional qualitative research. Further studies on supportive measures, such as ICT and health apps, are needed. In addition, it is very important to investigate the sustainability of back referrals and intervene on aspects that deviate from the intentions of the initial criteria. This could lead to changes in criteria or to extra interventions that support the referral back to primary care. Some stakeholders may ask for studies on the (cost-) effectiveness of back referrals.

### Strengths and limitations

To our knowledge, this is the first study in which the criteria for back referrals for patients with CKD have been explicitly formulated. We decided to take a regional approach by inviting eight nephrologists from different hospitals (general and academic) and two GPs, all from the southeast of the Netherlands, to identify the criteria for back referrals. This interdisciplinary approach was intended to ensure that these criteria resulted in a longer-lasting working relationship to provide the trust needed for a nephrologist to refer a patient back to primary care. The consensus process could have involved more than two GPs, but as the referral back should be initiated by nephrologists, we considered this to be an adequate balance. In addition, the recently developed transmural guideline supported the consensus process [32]. Other potential limitations exist in this work. We only reviewed the records of patients under nephrologist care, but patients with CKD who are treated by a general internist would likely also meet the BR criteria and could be included in future studies. We tried to avoid selection bias by instructing the nephrologists to invite all patients to participate in this study; nevertheless, we cannot fully exclude the possibility that selection bias may have occurred.

## Conclusion

Taking the starting point that patients with less-progressive moderate or even stable advanced CKD can be managed in primary care, we developed criteria for back referrals. When applying these BR criteria to patients with CKD in outpatient clinics, a substantial proportion of patients turned out to be eligible for referral back to primary care. These patients often had cardiovascular comorbidities, and their renal care could therefore constitute part of a chronic care programme managed by their GP. Given the nature of CKD, such a programme would require a strong shared-care identity, supported by consultations between GPs and nephrologists. Future research should focus on the feasibility of actually implementing referrals back to primary care, the follow up of renal function in such a setting, and patient satisfaction with care.

## Data Availability

The dataset analysed during the current study is available from the corresponding author on reasonable request.

## Abbreviations

ADPKD: Autosomal dominant polycystic kidney disease
COPD: Chronic obstructive pulmonary disease
CKD: Chronic kidney disease
CWH: Canisius Wilhelmina Hospital
eGFR: Estimated glomerular filtration rate
GP: General practitioner
HIV / AIDS: Human immunodeficiency virus / acquired immunodeficiency syndrome
BR criteria: Back referral criteria
RRT: Renal replacement therapy
RUMC: Radboud university medical center
SD: Standard deviation
TIA/CVA: Transient ischaemic accident / Cerebrovascular accident
VMC: VieCurie Medical Center

## References

[1] Hill NR, Fatoba ST, Oke JL, Hirst JA, O'Callaghan CA, Lasserson DS (2016). Global prevalence of chronic kidney disease - a systematic review and meta-analysis. PLoS One.

[2] Kidney Disease: Improving Global Outcomes (KDIGO) CKD Work Group. KDIGO (2012). Clinical practice guideline for the evaluation and Management of Chronic Kidney Disease. Kidney Inter Suppl.

[3] Nederlands huisartsen genootschap. NHG - Standaard Chronische Nierschade. Huisarts en Wetenschap; 2018.

[4] NICE Clinical guideline: Chronic kidney disease. Early identification and management of chronic kidney disease in adults in primary and secondary care. London: National Collaborating Centre And National Institute for Health and care Excellence; 2008 September (last modified 2014, July).

[5] Smart NA, Titus TT (2011). Outcomes of early versus late nephrology referral in chronic kidney disease: a systematic review. Am J Med.

[6] Gansevoort RT, Correa-Rotter R, Hemmelgarn BR, Jafar TH, Heerspink HJ, Mann JF (2013). Chronic kidney disease and cardiovascular risk: epidemiology, mechanisms, and prevention. Lancet..

[7] Coresh J, Selvin E, Stevens LA, Manzi J, Kusek JW, Eggers P, van Lente F, Levey AS (2007). Prevalence of chronic kidney disease in the United States. JAMA.

[8] Singh K, Waikar SS, Samal L (2017). Evaluating the feasibility of the KDIGO CKD referral recommendations. BMC Nephrol.

[9] Vanholder R, Annemans L, Brown E, Gansevoort R, Gout-Zwart JJ, Lameire N (2017). Reducing the costs of chronic kidney disease while delivering quality health care: a call to action. Nat Rev Nephrol.

[10] Coresh J (2017). Update on the burden of CKD. J Am Soc Nephrol.

[11] Castor EDC. https://www.castoredc.com [.

[12] Van Gelder VA, Scherpbier-De Haan ND, De Grauw WJ, Vervoort GM, Van Weel C, Biermans MC, et al. Quality of chronic kidney disease management in primary care: a retrospective study. Scand J Prim Health Care. 2016:1–8. 10.3109/02813432.2015.1132885 PMC4911031 26853071

[13] Allen AS, Forman JP, Orav EJ, Bates DW, Denker BM, Sequist TD (2011). Primary care management of chronic kidney disease. J Gen Intern Med.

[14] Meran S, Don K, Shah N, Donovan K, Riley S, Phillips AO (2011). Impact of chronic kidney disease management in primary care. QJM..

[15] Stevens KK, Woo YM, Rodger RS, Geddes CC (2009). Discharging patients from the nephrology clinic to primary care--will they get appropriate monitoring of renal function?. QJM..

[16] McClure M, Jorna T, Wilkinson L, Taylor J (2017). Elderly patients with chronic kidney disease: do they really need referral to the nephrology clinic?. Clin Kidney J.

[17] van Dipten C, van Berkel S, van Gelder VA, Wetzels JF, Akkermans RP, de Grauw WJ (2017). Adherence to chronic kidney disease guidelines in primary care patients is associated with comorbidity. Fam Pract.

[18] Samal L, Wright A, Waikar SS, Linder JA (2015). Nephrology co-management versus primary care solo management for early chronic kidney disease: a retrospective cross-sectional analysis. BMC Nephrol.

[19] Scherpbier-de Haan ND, Vervoort GM, van Weel C, Braspenning JC, Mulder J, Wetzels JF (2013). Effect of shared care on blood pressure in patients with chronic kidney disease: a cluster randomised controlled trial. Br J Gen Pract.

[20] Jones C, Roderick P, Harris S, Rogerson M (2006). An evaluation of a shared primary and secondary care nephrology service for managing patients with moderate to advanced CKD. Am J Kidney Dis.

[21] Dean J (2012). Organising care for people with diabetes and renal disease. J Ren Care.

[22] Major RW, Brown C, Shepherd D, Rogers S, Pickering W, Warwick GL, Barber S, Ashra NB, Morris T, Brunskill NJ (2019). The primary-secondary care partnership to improve outcomes in chronic kidney disease (PSP-CKD) study: a cluster randomized trial in primary care. J Am Soc Nephrol.

[23] van Gelder VA, Scherpbier ND, van Berkel S, Akkermans RP, De Grauw IS, Adang EM (2017). Web-based consultation between general practitioners and nephrologists, a cluster randomized controlled trial. Fam Pract.

[24] Haase A, Stracke S, Chenot JF, Weckmann G (2019). Nephrologists' perspectives on ambulatory care of patients with non-dialysis chronic kidney disease - a qualitative study. Health Soc Care Community.

[25] Garcia Garcia M, Valenzuela Mujica MP, Martinez Ocana JC, Otero Lopez Mdel S, Ponz Clemente E, Lopez Alba T (2011). results of a coordination and shared clinical information programme between primary care and nephrology. Nefrologia..

[26] Wilson C, Campbell SM, Luker KA, Caress AL. Referral and management options for patients with chronic kidney disease: perspectives of patients, generalists and specialists. Health Expect. 2015;18(3):325–34. 10.1111/hex.12025 PMC5060784 23216832

[27] Diamantidis CJ, Powe NR, Jaar BG, Greer RC, Troll MU, Boulware LE (2011). Primary care-specialist collaboration in the care of patients with chronic kidney disease. Clin J Am Soc Nephrol.

[28] Greer RC, Liu Y, Cavanaugh K, Diamantidis CJ, Estrella MM, Sperati CJ (2019). Primary care Physicians' perceived barriers to nephrology referral and co-management of patients with CKD: a qualitative study. J Gen Intern Med.

[29] Tonkin-Crine S, Santer M, Leydon GM, Murtagh FE, Farrington K, Caskey F (2015). GPs' views on managing advanced chronic kidney disease in primary care: a qualitative study. Br J Gen Pract.

[30] Smekal MD, Tam-Tham H, Finlay J, Donald M, Benterud E, Thomas C (2018). Perceived benefits and challenges of a risk-based approach to multidisciplinary chronic kidney disease care: a qualitative descriptive study. Can J Kidney Health Dis.

[31] Bhachu HK, Cockwell P, Subramanian A, Nirantharakumar K, Kyte D, Calvert M (2019). Cross-sectional observation study to investigate the impact of risk-based stratification on care pathways for patients with chronic kidney disease: protocol paper. BMJ Open.

[32] Federatie medisch specialisten. Multidisciplinaire richtlijn Chronische nierschade. 2018. https://richtlijnendatabase.nl/richtlijn/chronische_nierschade_cns/startpagina_-_chronische_nierschade_cns.html.

